# Narrative review of neoadjuvant therapy in patients with locally advanced colon cancer

**DOI:** 10.1002/kjm2.12926

**Published:** 2024-12-24

**Authors:** Jen‐Pin Chuang, Yen‐Chen Chen, Jaw‐Yuan Wang

**Affiliations:** ^1^ Chiayi Hospital Ministry of Health and Welfare Chiayi Taiwan; ^2^ Department of Surgery, Faculty of Medicine, College of Medicine National Cheng Kung University Tainan Taiwan; ^3^ Department of Surgery National Cheng Kung University Hospital Tainan Taiwan; ^4^ Division of Colorectal Surgery, Department of Surgery, Kaohsiung Medical University Hospital Kaohsiung Medical University Kaohsiung Taiwan; ^5^ Graduate Institute of Clinical Medicine, College of Medicine Kaohsiung Medical University Kaohsiung Taiwan; ^6^ Department of Surgery, Faculty of Medicine, College of Medicine Kaohsiung Medical University Kaohsiung Taiwan; ^7^ Graduate Institute of Medicine, College of Medicine Kaohsiung Medical University Kaohsiung Taiwan; ^8^ Center for Cancer Research Kaohsiung Medical University Kaohsiung Taiwan

**Keywords:** locally advanced colon cancer, mismatch repair, neoadjuvant chemoradiotherapy, neoadjuvant chemotherapy, neoadjuvant immunotherapy

## Abstract

Colorectal cancer is a leading cause of cancer‐related morbidity and mortality worldwide, with more than 1.9 million new cases reported in 2020, and is associated with major survival challenges, particularly in patients with locally advanced colon cancer (LACC). LACC often involves T4 invasion or extensive nodal involvement and requires a multidisciplinary approach for management. Radical surgery followed by adjuvant chemotherapy remains the primary treatment strategy for LACC. However, achieving complete tumor resection (R0) is challenging because locally advanced colon tumors typically infiltrate adjacent organs or nodes. Advancements in LACC treatment have involved neoadjuvant chemotherapy (NACT), neoadjuvant chemoradiotherapy (NACRT), and neoadjuvant immunotherapy (NAIT). Studies such as FOxTROT and PRODIGE 22 have demonstrated that NACT, particularly with FOLFOX or CAPOX, can lead to major tumor downstaging, improved survival rates, and increased R0 resection rates. Predictive biomarkers, such as mismatch repair (MMR) status and T stage, are crucial in identifying candidates who may benefit from NACT. NACRT has demonstrated promise in enhancing tumor regression, particularly in patients with rectal cancer, underscoring its potential for use with LACC. NAIT, particularly for deficient MMR tumors, has emerged as a novel approach, with studies such as NICHE‐2 and NICHE‐3 reporting excellent pathologic responses and pathologic complete responses. Integrating these therapies can enhance the surgical and survival outcomes of patients with LACC, highlighting the importance of personalized treatment strategies based on tumor characteristics and response to neoadjuvant interventions. This review discusses the evolving landscape of LACC management, focusing on optimizing treatment approaches for improved patient outcomes.

## 1 INTRODUCTION

For over a decade, colorectal cancer (CRC) has been the third most commonly diagnosed cancer in men and the second most commonly diagnosed cancer in women worldwide.^1^ According to the 2020 report of the World Health Organization's Global Cancer Observatory, CRC is the third most common type of cancer and the second leading cause of cancer‐related deaths worldwide for both men and women. In 2020, more than 1.9 million new cases of CRC were reported worldwide, with nearly 1,148,515 of these cases being colon cancer.^2^ Locally advanced colon cancer (LACC) refers to stage T4 colon cancer in which the tumor either invades nearby organs or has extensive regional lymph node involvement. This subtype of cancer accounts for ~10%–15% of all cases of advanced stage colon cancer and generally has a poor prognosis.^3^, ^4^, ^5^, ^6^ Approximately 5% of patients with colon cancer present at an advanced stage with locally unresectable malignancies resulting from major organ involvement or direct invasion.^7^ According to the *AJCC Cancer Staging Manual*, LACC occurs in two stages: stage IIB/C and stage IIIB/C. In contrast to the seventh edition of the manual, the eighth edition strongly emphasizes the adverse prognostic indicators associated with the extent of tumor invasion, despite a reduced number of positive lymph nodes.^8^, ^9^ The extent of invasion in LACC can be further divided into two substages: T4a, characterized by visceral peritoneum infiltration, and T4b, characterized by adjacent tissue or organ invasion.^9^, ^10^ According to the Surveillance, Epidemiology, and End Results database [9], patients with stage IIB (T4aN0M0) and stage IIC (T4bN0M0) colon cancer typically have 5‐year survival rates of 60.6% and 45.7%, respectively.^11^ Compared with patients with stage IIIA (T1‐2N1/T1N2a) colon cancer, those with stage IIB or IIC have a poorer prognosis. Patients with stage IIC, IIIB, and IIIC LACC have 5‐year survival rates of 37.3%, 46.3%, and 28%, respectively, indicating a lower 5‐year survival rate in stage IIC than in stage IIIB colon cancer [7]. In patients with node‐positive T4 (stage IIIC), the 5‐year survival rate decreases to <40%, which is similar to the survival rate of patients at stage IV.^11^, ^12^

The primary treatment modality for LACC is radical surgery followed by adjuvant chemotherapy, as established by standard practice.^3^, ^6^, ^13^, ^14^, ^15^, ^16^, ^17^ Managing patients with LACC represents a surgical challenge because their lesions often extend into adjacent organs and because regional lymph node metastasis often affects the root of the main feeding artery.^18^, ^19^ In cases requiring curative surgery, en bloc multivisceral resection is essential to ensure adequate resection margins.^3^, ^20^, ^21^ However, certain risk factors, such as cT4, cN+ status, and poor or undifferentiated pathology in LACC, reduce the likelihood of achieving R0 resection.^5^, ^22^ These challenges have prompted research into new treatment modalities for LACC aimed at addressing the difficulties posed by tumor invasion into adjacent organs or extensive lymph node metastasis.

Neoadjuvant chemotherapy (NACT) and neoadjuvant chemoradiotherapy (NACRT) have the potential to promote tumor reduction, eliminate micrometastases, and prevent tumor cell shedding during surgical procedures for the management of patients with advanced gastrointestinal tumors.^23^, ^24^, ^25^, ^26^, ^27^ Many studies have explored the effectiveness and safety of NACT, particularly in the context of LACC.^18^, ^28^, ^29^, ^30^, ^31^, ^32^, ^33^, ^34^, ^35^, ^36^ Other studies have specifically examined the oncological benefits of neoadjuvant FOLFOX therapy and anti‐epidermal growth factor receptor treatment in managing patients with LACC.^28^, ^31^, ^32^ These studies have indicated that combining NACT with a FOLFOX regimen can achieve major tumor downstaging while maintaining acceptable toxicity levels.^30^, ^31^, ^35^ To achieve these outcomes, the National Comprehensive Cancer Network recommends the use of NACT in patients with colon cancer who have bulky nodal disease or cT4 status.^37^ Chalabi et al.^33^, ^38^ reported promising outcomes of neoadjuvant immunotherapy (NAIT) in patients with LACC with mismatch repair (dMMR) status, particularly in terms of disease downstaging. These findings indicate that neoadjuvant therapy has become a key component of optimizing treatment for LACC. The current review explored emerging trends and recent advancements in this field, offering a comprehensive overview of strategies aimed at improving the outcomes of patients with LACC.

## 2 MATERIALS AND METHODS

A comprehensive review was conducted to identify studies exploring the role of neoadjuvant therapy in the management of patients with LACC. This review involved an extensive search across multiple databases, including PubMed, Cochrane Review, Cochrane Central Register of Controlled Trials, and ClinicalTrials.gov, from the earliest available records until August 2024. No restrictions were imposed regarding language or geographical region during the search process. The search terms used included “colon cancer,” “locally advanced,” “neoadjuvant chemotherapy,” “neoadjuvant chemoradiotherapy,” “neoadjuvant immunotherapy,” “downstaging,” “Eoverall survival (OS),” and “disease‐free survival (DFS).” Additionally, a comprehensive manual review of bibliographies and relevant studies was conducted to identify any additional potentially eligible studies.

## 3 ROLE OF NACT AND NACRT IN LACC TREATMENT

### 3.1 Regimens and protocols of NACT for LACC

FOxTROT (NCT00647530) is the largest prospective, multicenter, randomized clinical trial conducted to evaluate the efficacy of NACT in cases of radiologically confirmed LACC characterized as cT3 (with an invasive growth of 5 mm or more beyond the muscularis propria) or cT4 (in which the tumor extends to the surface of the visceral peritoneum or beyond).^35^, ^36^, ^39^ In this trial, 1053 patients were randomly divided into 2 groups at a ratio of 2:1. The first group received neoadjuvant FOLFOX treatment for 6 weeks, which consisted of 5‐fluorouracil, leucovorin, and oxaliplatin, with an addition of panitumumab, depending on the patient's *RAS* wild‐type status, followed by surgical resection and adjuvant chemotherapy. The second group underwent surgical resection followed by 24 weeks of systemic therapy in an adjuvant setting.^35^ PRODIGE 22 is an ongoing French clinical trial designed to explore different treatment strategies for patients with resectable high‐risk T3, T4, and N2 CRC, with the strategy selected on the basis of their baseline computed tomography (CT) scans. In this trial, 120 patients were randomly assigned to receive either 6 months of adjuvant FOLFOX following colectomy (control group) or perioperative FOLFOX, consisting of four cycles before surgery and eight cycles after surgery (perioperative group). A third group of 16 patients with *RAS* wild‐type tumors was included to evaluate the efficacy of perioperative FOLFOX combined with cetuximab. However, the FOLFOX‐cetuximab arm was discontinued after interim analysis because of a lack of efficacy.^31^, ^36^, ^40^ The OPTICAL trial is a Chinese trial involving 744 patients with LACC (T4 or T3 with ≥5 mm invasion beyond the muscularis propria). In this trial, patients were randomly assigned at a ratio of 1:1 to either receive 3 months of NACT with mFOLFOX6 or CAPOX followed by surgery and 3 months of adjuvant chemotherapy (experimental group) or to undergo upfront surgery with optional adjuvant chemotherapy, administered on the basis of their pathological stage at the investigator's discretion (standard care group).^41^ The Scandinavian NeoCol trial is a randomized, controlled, phase III study conducted across nine centers in three countries. This trial involved 248 patients with biopsy‐confirmed LACC (T4 or T3 with ≥5 mm invasion, N0‐2, M0). These patients were randomly assigned to either undergo standard upfront surgery (122 patients) or receive NACT followed by surgery (126 patients). The neoadjuvant group received either three cycles of CAPOX or four cycles of FOLFOX before surgery, and adjuvant chemotherapy was initiated on the basis of each patient's pathological stage, following clinical guidelines. Finally, the outcomes of the two treatment strategies were compared.^42^

NACRT has been widely used in patients with locally advanced rectal cancer because of its positive effect on tumor regression.^23^, ^25^ Multiple studies have explored the oncological benefits of NACRT for patients with LACC, with cumulative evidence indicating that NACRT followed by surgery is an effective treatment modality for these patients.^4^, ^43^, ^44^, ^45^ In these studies, the patients simultaneously received radiotherapy and mFOLFOX6 chemotherapy every 2 weeks. Pretreatment CT scanning was used to define each patient's gross tumor volume and clinical target volume, with a margin of 15–20 mm incorporated. Regardless of each patient's tumor location (left or right), a radiation dose of 45 Gy was delivered in 25 fractions to the lymphatic regions, followed by a 50 Gy boost to the primary tumor. The volume of the small bowel receiving more than 50 Gy of radiation was limited to <1 cc, and the maximum dose delivered to the spinal cord was limited to under 45 Gy.^4^

### 3.2 Oncological benefits of NACT and NACRT for patients with LACC

Preliminary findings from the FOxTROT trial have demonstrated the oncological safety and potential benefits of NACT for patients with LACC. These benefits include improved survival outcomes, increased rates of R0 resection, and a major reduction in treatment‐related toxicity and perioperative complications, which were observed in the majority of cases.^35^ A notable finding was that of a strong association between the pathologic tumor regression score (Dworak score) and the risk of recurrence. Patients who achieved a complete pathologic response (3.5%) experienced no recurrence within 5 years, whereas those who did not experience tumor regression (35%) had a 29% risk of recurrence over the same period. The results also revealed a 4.6% absolute reduction in residual or recurrent disease at 2 years with preoperative therapy (16.9% vs 21.5%), with a rate ratio (RR) of 0.72 and a 95% confidence interval (CI) of 0.54–0.98 (*p* = 0.037). A similar decreasing trend was observed in colon cancer‐specific mortality (RR = 0.74, 95% CI: 0.52–1.05, *p* = 0.095) and all‐cause mortality (RR = 0.76, 95% CI = 0.55–1.06, *p* = 0.104), although these results did not reach statistical significance. Compared with their counterparts, patients who received preoperative therapy were more likely to undergo an R0 resection.^46^, ^47^ Subsequent analysis revealed significantly longer overall survival (OS) in patients with high epiregulin and amphiregulin expression who received treatment with panitumumab combined with FOLFOX for 6 weeks compared with those receiving FOLFOX alone.^48^

In the PRODIGE 22 trial, perioperative FOLFOX chemotherapy was discovered to be associated with a significant pathological tumor regression grade (44% for grade 1 vs 8% for grade 2, *p* < 0.001) and a trend toward tumor downstaging in patients with high‐risk stage II and III colon cancer. However, no significant difference was observed in survival outcomes when perioperative FOLFOX chemotherapy was used.^31^, ^40^

In the OPTICAL trial, preoperative therapy was discovered to be associated with a 2% improvement in the primary endpoint of 3‐year disease‐free survival (DFS), although this difference was not significant (78.7% vs 76.6%, hazard ratio [HR] = 0.83, 95% CI: 0.60–1.15, *p* = 0.138). By contrast, it was found to be associated with a significant improvement in 3‐year OS (94.9% vs 88.5%, HR = 0.43, 95% CI: 0.22–0.83, *p* = 0.012). The survival curves began to separate at nearly 20 months and continued to diverge over 5 years. In addition, preoperative therapy significantly improved the DFS of women, with a rate of 84.2% compared with that of 74.7% in men (HR = 0.54, 95% CI: 0.31–0.93, *p* = 0.02).^41^

In the Scandinavian NeoCol trial, delivering three cycles of neoadjuvant CAPOX was discovered to be associated with a smaller effect on pT4 disease compared with initial surgery (28% vs 32%), with a complete response achieved in 3% of the cases. Node‐negative disease was more frequent (59% vs 48%) and lymphovascular invasion was less common (25% vs 39%) in the preoperative group than in the control group. In addition, the R0 resection rate was slightly higher in the neoadjuvant group than in the control group (93% vs 90%), although no significant difference was observed in the 5‐year DFS rate, which was 85% in the two groups.^42^

In 2020, a comprehensive meta‐analysis involving 29,504 patients revealed that NACT was associated with significant improvements in both OS (HR = 0.76, 95% CI: 0.65–0.89, *p* = 0.0005) and DFS (HR = 0.74, 95% CI: 0.58–0.95, *p* = 0.02) in patients with LACC. These benefits were achieved without increasing surgical complications, which differ from outcomes of the conventional approach of initiating treatment with surgery followed by chemotherapy.^49^ A meta‐analysis conducted in 2023, which included a larger cohort of 31,047 patients with LACC, reported similar positive outcomes in terms of OS after treatment with NACT but reported no significant difference in DFS compared with that associated with other treatment strategies.^50^

In our previous study, we reported that, in 34 patients who received NACRT followed by surgery, the rate of pathologic complete responses was 26.4%, and the rate of R0 resection was 91.2%.^51^ Multiple studies with small sample sizes have indicated that NACRT is a feasible and safe option for patients with LACC.^43^, ^44^, ^45^, ^51^ In a single‐institute observational study of 100 patients with unresectable LACC who received NACRT, R0 resection was achieved in 83 patients, with grade 3 or 4 myelosuppression observed in only 17 patients.^45^

### 3.3 Predictive biomarkers of LACC in patients receiving NACT

#### 3.3.1 dMMR versus proficient MMR

Tumors characterized by a loss of MMR proteins are classified as dMMR tumors, whereas those with intact MMR proteins are classified as proficient MMR (pMMR) tumors. Typically, dMMR tumors produce truncated, nonfunctional proteins or lack MMR proteins altogether, which can contribute to their oncogenesis. Normally, the ratio of dMMR in sporadic CRC and stage III colon cancer is ~15% and 12%, respectively.^52^, ^53^ In the FOxTROT trial, NACT was reported to result in moderate or high histological tumor regression in 23% of patients with pMMR tumors but in only 7% of patients with dMMR tumors (*p* < 0.001).^32^ Only patients with pMMR tumors experienced a significant reduction in the risk of relapse at 2 years, with 16.8% of those with pMMR tumors exhibiting a complete or marked response. Compared with patients with pMMR tumors, those with dMMR tumors exhibited higher resistance to chemotherapy, with 51.2% of those with dMMR tumors demonstrating a poor or no response and only 25.7% of those with pMMR tumors demonstrating a poor or no response.^40^, ^41^ A retrospective study published in 2022 reported conflicting findings. Specifically, among 83 patients with cT4 colon cancer, the majority had a mild (dMMR vs pMMR: 64.5% vs 47.6%) or moderate (dMMR vs pMMR: 16.1% vs 28.6%) tumor regression grade. More than half of the patients with dMMR tumors experienced disease downstaging (64.5% in patients with dMMR tumors and 47.6% in patients with pMMR tumors). These results confirmed the efficacy of neoadjuvant chemotherapy in patients with advanced dMMR tumors and highlighted the potential capability of this chemotherapeutic regimen to reduce the rate of multiorgan resection in patients with cT4b dMMR tumors.^54^

#### 3.3.2 T stage

According to current clinical guidelines, preoperative chemotherapy is recommended for patients with cT4 colon cancer; this is likely because little high‐quality evidence has been reported in the literature.^37^, ^55^ A nationwide study reported survival benefits to be primarily associated with NACT in patients with T4b LACC, not in those with T3 or T4a LACC. This finding suggests that T4b is a key factor indicating the potential for tumor shrinkage with NACT.^56^

#### 3.3.3 Excision repair cross‐complementing group 1, thymidylate synthase, and glutathione S‐transferase

In addition to MMR status, the expression of excision repair cross‐complementing group 1 (ERCC1) has attracted attention because of its major role in the repair of platinum‐induced DNA damage. This DNA excision repair protein participates in DNA repair and recombination in human cells. In a study involving 70 patients with advanced CRC, the expression levels of ERCC1 and thymidylate synthase (TS) were examined as potential negative prognostic factors for FOLFOX‐based NACT.^57^ Another study involving 39 patients with advanced CRC reported that patients without ERCC1 or glutathione S‐transferase (P1) expression but with TS expression were likely to respond to FOLFOX chemotherapy.^58^ Another study revealed that the overexpression of ERCC1 was associated with a poor response to cT4b CRC with FOLFOX‐based NACT.^59^

## 4 ROLE OF NAIT IN LACC TREATMENT

### 4.1 Regimens and protocols of NAIT for LACC

In 2024, Chalabi et al.^38^ published a phase 2 study on the impact of NAIT on patients with dMMR LACC. The study involved 115 patients with cT3 or N+ dMMR colon cancer who received nivolumab (3 mg/kg) and ipilimumab (1 mg/kg), both administered on day 1, with a second dose of nivolumab administered on day 15. The primary endpoints were safety, defined as timely surgery (no more than a 2‐week delay due to toxicity), and 3‐year DFS. The secondary endpoints included pathologic responses and genomic analyses. In the NICHE‐3 study, 19 patients with resectable dMMR LACC received two doses of nivolumab (480 mg) and relatlimab (480 mg) at 4‐week intervals, followed by surgery within 8 weeks.^60^ The primary endpoint was the pathologic response rate.

### 4.2 Oncological outcomes of patients with LACC receiving NAIT

In the NICHE‐2 study, which involved 115 patients with dMMR LACC, 98% of the patients underwent timely surgery, with only 2 patients experiencing delays. Grade 3 or 4 immune‐related adverse events (irAEs) occurred in 4% of the patients, but none of them required treatment discontinuation because of these events. A pathologic response was observed in 98% of the patients, with a major pathologic response and a complete response observed in 95% and 68% of the patients, respectively, and no disease recurrence observed after a median follow‐up of 26 months.^38^ In the NICHE‐3 study, which involved 19 patients, 74% of the patients experienced mild irAEs, predominantly infusion reactions, with cases of severe irAEs being rare. Treatment was discovered to be associated with a pathologic complete response of 79%, a major pathologic response of 89%, and an overall pathologic response rate of 100%.^60^

### 4.3 Predictive biomarkers of LACC in patients receiving NAIT

**FIGURE 1:**
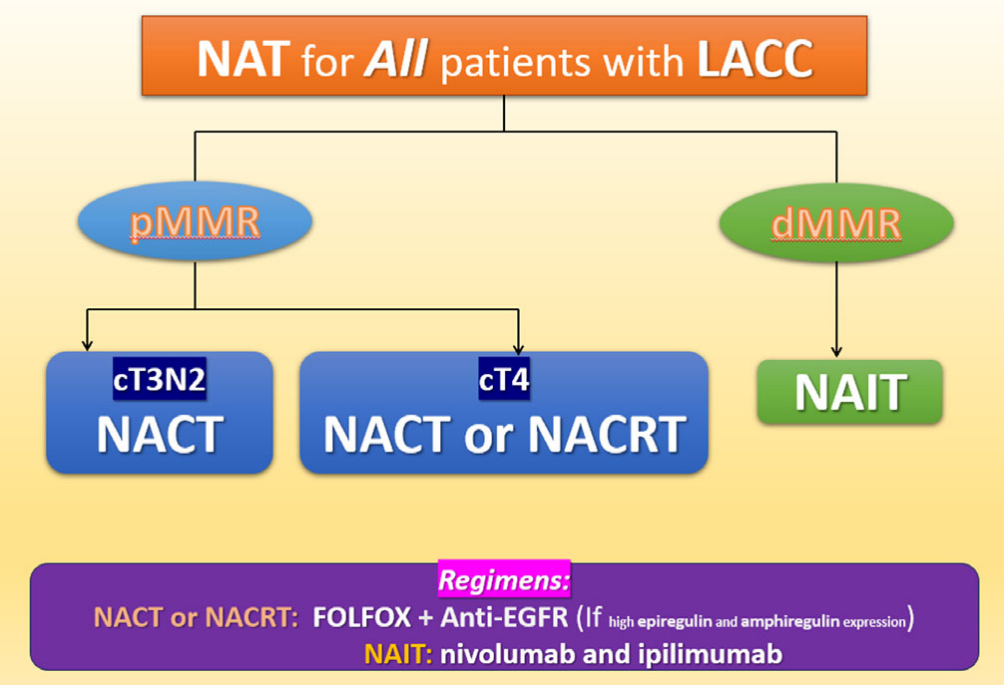
Strategy of NAT for LACC.

MMR status plays a key role in the effectiveness of immunotherapy against colon cancer. Compared with patients with pMMR tumors, those with dMMR tumors have a more favorable prognosis but tend to be less responsive to chemotherapy. Typically, patients with dMMR CRC have a significantly large number of neoantigens, and therefore, they are responsive to immune checkpoint inhibitors.^61^, ^62^, ^63^ In a study involving 35 patients with early‐stage colon cancer treated with a combination of ipilimumab and nivolumab, all 20 patients in the dMMR group exhibited a pathologic response (100%, 95% CI: 86%–100%), whereas only 4 out of 15 patients in the pMMR group (27%) demonstrated a response.^64^ Immune checkpoint inhibitors have been associated with promising results in patients with metastatic CRC belonging to the dMMR or microsatellite instability‐high (MSI‐H) subgroup.^65^, ^66^ However, the mechanisms underlying tumor mutation burden, programmed death ligand 1 expression, and polymerase epsilon in patients with metastatic CRC undergoing immunotherapy must be further elucidated (Figure 1; Table 1).^67^

**TABLE 1:** The role of NACT, NACRT, and NAIT in LACC.

Treatment	Regimens and protocols	Oncological outcomes	Predictive biomarkers
NACT	FOxTROT: 6 weeks of neoadjuvant FOLFOX with or without panitumumab based on RAS status, followed by surgery and adjuvant chemotherapy^35^, ^39^	Improved survival outcomes, R0 resection (94%), and reduced recurrence^35^, ^46^, ^48^, ^77^	MMR status: dMMR tumors showed less response to chemotherapy compared to pMMR, with pMMR achieving significant reduction in recurrence^32^ T stage T4b with better survival benefits of NACT^56^ ERCC1 expression negatively impacted FOLFOX response^57^, ^59^
	PRODIGE 22: Perioperative FOLFOX with or without cetuximab for high‐risk cases^31^, ^36^, ^40^	Significant pathological tumor regression^31^, ^40^	
	OPTICAL: 3 months of neoadjuvant mFOLFOX6 or CAPOX, then surgery, followed by adjuvant chemotherapy^41^	Improved 3‐year OS (94.9% vs 88.5%) and DFS benefit in women^41^	
	NeoCol: CAPOX or FOLFOX neoadjuvant cycles before surgery^42^	Modest response with node‐negative disease in 59% of cases and 85% DFS^42^	
NACRT	Radiotherapy combined with mFOLFOX6, with dose adjusted by tumor location (left‐ or right‐sided). CT planning for tumor volume, with margins for lymph nodes and target volume^4^, ^43^, ^44^, ^45^, ^51^	Pathological complete response rate up to 26.4% and R0 resection rate of 91.2%^51^	Predictive biomarker studies were limited, but efficacy for tumor regression across various genetic profiles
		Single‐institute study: R0 achieved in 83% of unresectable cases; some toxicity with myelosuppression in 17%^45^	
NAIT	NICHE‐2: Nivolumab and ipilimumab regimens for dMMR LACC, followed by surgery^38^	98% pathologic response, with 95% major and 68% complete response, and no recurrence at median 26 months^38^	MMR status: dMMR/MSI‐H tumors highly responsive to immune checkpoint inhibitors^38^, ^61^, ^64^ Tumor mutation burden and PD‐L1 expression positively correlated with response to immunotherapy^67^
	NICHE‐3: Nivolumab and relatlimab for dMMR cases, surgery after two doses^60^	100% overall pathologic response, 79% complete response^60^	

## 5 DISCUSSION

NACT offers several advantages over traditional postoperative treatment. Administering chemotherapy before surgery enables earlier targeting of micrometastases. This approach also allows for more flexibility in treatment, including allowing for therapies to be switched early or organ‐sparing techniques to be utilized when appropriate. One of the benefits of neoadjuvant therapy is its lower toxicity profile compared with that of adjuvant chemotherapy,^42^ which may reduce side effects, promote patient compliance, and improve treatment delivery rates.^68^ In a previous study, the rates of sensory and motor neuropathy were found to decrease during both treatment and follow‐up in patients receiving neoadjuvant therapy (9% vs 13% for sensory neuropathy and 3% vs 8% for motor neuropathy).^42^ These findings are likely attributable to the chemotherapy break during surgery and the reduced number of cycles required.

Patients with advanced disease, such as those with T4 or extensive node‐positive tumors, may substantially benefit from neoadjuvant therapy, given their low survival rates despite undergoing standard postoperative treatments.^69^, ^70^ However, preoperative staging of colon cancer through CT scans often leads to overtreatment, with 24%–33% of patients being inaccurately classified into higher‐risk categories, resulting in unnecessary treatments for those with stage I or low‐risk stage II disease.^36^, ^39^ Consistent with these findings, the OPTICAL trial in China revealed a tendency toward overtreatment, with many patients later classified into low‐risk stage II after upfront surgery.^41^ Taken together, these findings underscore the need for improved radiologic criteria for more precise identification of patients with high‐risk stage II and III colon cancer. Enhancing the CT scanning criteria or exploring novel imaging modalities such as magnetic resonance imaging, which has demonstrated effectiveness in rectal cancer, may address these challenges.

Multiple clinical studies have examined the role of neoadjuvant therapy in the treatment of LACC. For example, the FOxTROT trial, which was a pioneering trial, demonstrated the safety and feasibility of NACT in patients with colon cancer, although only a small percentage (7%) of patients with dMMR tumors exhibited a pathologic response.^39^ Immunotherapy has demonstrated promise in the treatment of patients with dMMR/MSI‐H tumors, as evidenced by the trial of Chalabi et al., in which patients with locally advanced tumors responded well to treatment.^33^, ^38^ Notably, conflicting results were reported by the NICOLE trial, in which no major pathologic responses were observed in the small subset of patients with dMMR tumors who received neoadjuvant nivolumab.^71^

Many dMMR/MSI‐H tumors are linked to Lynch syndrome (LS), a hereditary condition that increases the risk of multiple cancers. Early diagnosis of LS can help reduce the rate of cancer‐specific mortality by enabling early surveillance and intervention for secondary cancers.^72^, ^73^ Universal MMR testing is recommended by the National Institute for Health and Care Excellence and the National Comprehensive Cancer Network for patients with CRC to improve their diagnosis rates and outcomes.^73^ Over the past 3 decades, many biomarkers have been identified in tumor, stool, and tissue fluid specimens that have the potential to aid in the diagnosis and prognosis of colon cancer.^74^, ^75^, ^76^ However, many of these biomarkers only facilitate patient prognoses and do not have predictive value, which limits their ability to guide treatment strategies for patients with stage III colon cancer.^16^, ^57^, ^77^, ^78^, ^79^, ^80^, ^81^, ^82^ Therefore, further research is required to identify biomarkers that can accurately predict responses to neoadjuvant therapies and that therefore have the potential to transform the management of LACC.

## 6 CONCLUSION

Neoadjuvant therapy is a promising approach capable of improving the outcomes of patients with LACC. Findings from the FOxTROT trial continue to elucidate the therapeutic benefits of neoadjuvant therapy. Neoadjuvant radiotherapy has demonstrated major potential in tumor downstaging, particularly in specific LACC cases. In patients with cT4b dMMR LACC, NAIT is recommended when no contraindications are present. This strategy enables early intervention, reduces treatment toxicity, and allows for greater flexibility in the modification of treatment plans. However, challenges pertaining to accurate staging, patient compliance, and variability in immunotherapeutic responses remain, indicating a need for continual research and innovation. Advancements in imaging techniques, biomarker development, and personalized treatment approaches can facilitate the optimization of care for patients with colon cancer.

## CONFLICT OF INTEREST STATEMENT

All authors declare no conflicts of interest.

## Data Availability

The data that support the findings of this study are available on request from the corresponding author. The data are not publicly available due to privacy or ethical restrictions.
